# Supplementary material for: Effectiveness of utilizing the WHO safe childbirth checklist on improving essential childbirth practices and maternal and perinatal outcome: A systematic review and meta-analysis
Source: PLoS One. 2020 Jun 12;15(6):e0234320. doi: 10.1371/journal.pone.0234320 (PMC7292415; doi:10.1371/journal.pone.0234320)
Supplement: S1 Document — It indicates excluded studies and reasons for their exclusion. (DOCX) [file pone.0234320.s001.docx]

Studies excluded on the full text

Delaney MM, Maji P, Kalita T, Kara N, Rana D, Kumar K, et al. Improving adherence to essential birth practices using the WHO safe childbirth checklist with peer coaching: experience from 60 public health facilities in Uttar Pradesh, India. Global Health: Science and Practice. 2017;5(2):217-31.

***Reason for exclusion*:** reported the impact of peer coaching on adherence to WHO SCC and didn't report the outcome of interest.

Kara N, Firestone R, Kalita T, Gawande AA, Kumar V, Kodkany B, et al. The BetterBirth Program: Pursuing Effective Adoption and Sustained Use of the WHO Safe Childbirth Checklist Through Coaching-Based Implementation in Uttar Pradesh, India. Global health, science, and practice. 2017;5(2):232-43.

***Reason for exclusion*:** reported the impact of peer coaching on adherence to WHO SCC and didn't report the outcome of interest.

Patabendige M, Senanayake H. Implementation of the WHO safe childbirth checklist program at a tertiary care setting in Sri Lanka: a developing country experience. BMC pregnancy and childbirth. 2015; 15:12.

***Reason for exclusion*:** reported impact of Sri-Lanka context-specific modified WHO Safe Childbirth Checklist on adherence to WHO SCC and didn't report the outcome of interest.

Senanayake HM, Patabendige M. Piloting of WHO Safe Childbirth Checklist using a modified version in Sri Lanka. 2018;11(1):896.

***Reason for exclusion:*** Cross-sectional study without a comparative group (non-comparative nature of the study).
