# Supplementary material for: Effectiveness of utilizing the WHO safe childbirth checklist on improving essential childbirth practices and maternal and perinatal outcome: A systematic review and meta-analysis
Source: PLoS One. 2020 Jun 12;15(6):e0234320. doi: 10.1371/journal.pone.0234320 (PMC7292415; doi:10.1371/journal.pone.0234320)
Supplement: S1 Table — It indicates a detailed search strategy for PubMed. (DOCX) [file pone.0234320.s002.docx]

| Number | Query. | Result. |
| --- | --- | --- |
| 1 | (WHO[All Fields] AND ("checklist"[MeSH Terms] OR "checklist"[All Fields])) OR ("Practice (Birm)"[Journal] OR "practice"[All Fields]) AND ("delivery, obstetric"[MeSH Terms] OR ("delivery"[All Fields] AND "obstetric"[All Fields]) OR "obstetric delivery"[All Fields] OR "delivery"[All Fields]) OR ("parturition"[MeSH Terms] OR "parturition"[All Fields] OR "childbirth"[All Fields]) OR ("labour"[All Fields] OR "work"[MeSH Terms] OR "work"[All Fields] OR "labor"[All Fields] OR "labor, obstetric"[MeSH Terms] OR ("labor"[All Fields] AND "obstetric"[All Fields]) OR "obstetric labor"[All Fields]) OR ("labour"[All Fields] OR "work"[MeSH Terms] OR "work"[All Fields] OR "labor"[All Fields] OR "labor, obstetric"[MeSH Terms] OR ("labor"[All Fields] AND "obstetric"[All Fields]) OR "obstetric labor"[All Fields]) AND ("stillbirth"[MeSH Terms] OR "stillbirth"[All Fields] OR ("still"[All Fields] AND "birth"[All Fields]) OR "still birth"[All Fields]) | 4339 |
| 2 | Limit 1 to English AND clinical trials OR Randomized controlled trial OR Comparative study OR observation study AND ten years. | 241 |

Supporting information.

Appendix I: Search strategy

A search conducted on November 11/2019, MEDLINE (Ovid).
